# Enhancement of Non-Enzymatic Antioxidants in *Eutrema salsugineum* Under Salt Stress Depends on Salicylate Depletion

**DOI:** 10.3390/ijms27031168

**Published:** 2026-01-23

**Authors:** Ya-Jian Fang, Xin-Yue Yang, Lin-Bei Xie, Zhong-Wei Zhang, Shu Yuan

**Affiliations:** International Science and Technology Cooperation Base for Efficient Utilization of Nutrient Resources and Fertilizer Innovation, College of Resources, Sichuan Agricultural University, Chengdu 611130, China; tempesstissimo@163.com (Y.-J.F.); yang16970319@163.com (X.-Y.Y.); kkskado@163.com (L.-B.X.); zzwzhang@126.com (Z.-W.Z.)

**Keywords:** salicylic acid, *Eutrema*, *Arabidopsis*, salt stress, ascorbic acid, glutathione

## Abstract

*Eutrema salsugineum* is a model species for studying stress resistance, particularly extreme salinity, and is often compared with *Arabidopsis thaliana*. Previous research has shown that basal salicylic acid (SA) levels are significantly lower in *E. salsugineum* than in *A. thaliana*. In this study, subtractive hybridization revealed that SA-related genes were extensively induced in *Arabidopsis* but not in *Eutrema*. Using exogenous SA and the biosynthesis inhibitor paclobutrazol (PBZ), we further demonstrated that the low endogenous SA level in *Eutrema* significantly upregulates dehydroascorbate reductase (DHAR) and glutathione reductase (GR) gene expression, doubling the pools of total ascorbic acid and total glutathione. While SA treatment decreased the ratios of reduced ascorbic acid (ASA) to dehydroascorbate (DHA) and reduced glutathione (GSH) to oxidized glutathione (GSSG), PBZ treatment increased them, correspondingly modulating DHAR and GR activities and gene expression. The resulting enhancement of these key non-enzymatic antioxidants is a critical mechanism underpinning the superior salt tolerance of *Eutrema*.

## 1. Introduction

Soil salinity acts as a critical environmental constraint, suppressing crop yield and compromising seedling survival, which collectively endangers food security worldwide [1,2]. This form of stress adversely affects an estimated 20% of total arable land and 30% of irrigated farmland across the globe [3]. The capacity for salt tolerance is a complex trait that shows significant variation between plant species and among cultivars, further modulated by the organism’s developmental status, prevailing growth conditions, and specific agronomic practices [1,2,3].

Salt exposure triggers a biphasic plant response. The initial osmotic stress phase (Stage I) rapidly inhibits new leaf growth within hours, followed by an ionic stress phase (Stage II) that disrupts chlorophyll and accelerates leaf senescence [1,2,3]. Under saline conditions, reduced water uptake leads to the accumulation of sodium and chloride ions within cells, severely impairing physiological and metabolic processes [1,2,3]. The toxic accumulation of Na^+^ and Cl^−^ ions further inhibits photosynthesis by disrupting enzyme activity and pigment synthesis [4]. Subsequent ion exchange alterations elevate reactive oxygen and nitrogen species (ROS/RNS) while reducing stomatal conductance [5]. Consequently, maintaining cellular ion homeostasis, particularly cytoplasmic Na^+^ concentration, is critical for physiological function and survival. Salt stress ultimately impacts development across all stages, from root and shoot growth to flowering and senescence. In response, plants activate downstream signaling pathways to mitigate these effects [1,2,3,4,5].

Research over the past few decades has established *Eutrema salsugineum* as a key model for studying stress resistance due to its remarkable tolerance to salinity, cold, drought, and ozone, with a genome that is highly comparable to *Arabidopsis thaliana* [6,7]. Transcriptomic analyses have identified the molecular basis of this high stress tolerance, revealing that many stress-associated genes in *E. salsugineum*, including those related to abscisic acid (ABA) biosynthesis and signaling, are constitutively expressed at higher levels than in *A. thaliana* even under non-stress conditions [8,9,10,11]. Unlike *A. thaliana*, which shows substantial transcriptional activation upon stress exposure, *E. salsugineum* exhibits only minor changes in gene expression [8,9,10,11]. This pattern is mirrored at the protein level, where salt stress induces more pronounced changes in protein abundance in *Arabidopsis* than in *Eutrema* [12]. Together, these findings point to a “stress preparedness” strategy in *E. salsugineum* that underlies its halophytic nature. Further supporting this, metabolite profiling shows significant differences between the two species following salt stress [13], suggesting that metabolic adjustment and the activation of pre-existing enzymes provide a faster and more efficient response than synthesizing new proteins [14].

Research has shown a contrasting SA response to salt stress between *A. thaliana* and *E. salsugineum* [15]. Under control conditions, *E. salsugineum* SA concentrations were much lower than in *A. thaliana*, and NaCl treatment further reduced them in *E. salsugineum*, whereas it increased SA in *A. thaliana* [15]. Correspondingly, salt stress induced enhanced levels of reduced ascorbate and glutathione in *Eutrema*, but not in *Arabidopsis* [16]. The wetland halophyte plant species *Kosteletzkya pentacarpos* also showed depletion of salicylic acid with increased ascorbate contents under a mixed Cd + Zn treatment [17].

The functional importance of this SA reduction in *E. salsugineum* is not well understood, and the detailed mechanism through which SA depletion regulates non-enzymatic antioxidants needs further investigation. In the present work, subtractive hybridization indicated a broad induction of SA-related genes in *Arabidopsis* but not in *Eutrema*. Experiments using applied SA and an inhibitor confirmed that the inherent SA depletion in *Eutrema* significantly boosts the expression of *DHAR1* and *GR1* genes, doubling the concentrations of reduced ascorbic acid and glutathione. This fortified antioxidant capacity is identified as an important factor in the salt tolerance of *Eutrema*.

## 2. Results

### 2.1. Salt Stress-Responsive Genes Identified by Two-Round SSH in A. thaliana and E. salsugineum

Earlier microarray studies have shown that thousands of *Arabidopsis* genes—approximately 30% of the transcriptome—respond to salt stress [18]. To identify a more focused set of key genes involved in salt tolerance in *A. thaliana* and *E. salsugineum*, we conducted a two-round suppression–subtractive hybridization (SSH) screen [19,20] using RNA samples collected before and after NaCl treatment. Pairwise comparison of two rounds of SSH revealed that genes encoding SA-signaling pathogenesis-related proteins PR-1 and PR-5, as well as salicylate/benzoate carboxyl methyltransferase (SAMT), were prominently enriched in *Arabidopsis* (Table 1). In contrast, genes encoding NPK1-related protein kinase 2 (chlorate/nitrate transporter CHL1), WRKY transcription factor 46 (WRKY46), C2H2 zinc finger superfamily protein (C2H2), Plant Cadmium Resistance 2 (PCR2), and potassium transporter 10 (POT10) were prominently enriched in *Eutrema* (Table 2). All five genes are correlated with ionic homeostasis directly or indirectly.

### 2.2. SA Doubles in A. thaliana but Not in E. salsugineum Under Salt Stress

**Figure 1:**
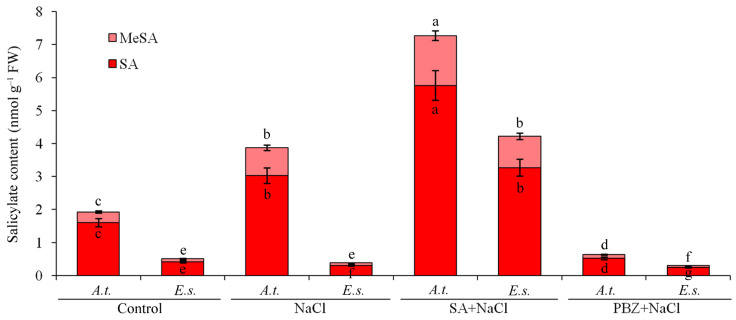
The effects of SA, PBZ, and NaCl treatments on endogenous SA and MeSA levels. For salt stress induction, 21-day-old seedlings were transferred to a half-strength MS nutrient solution supplemented with 200 mM NaCl for three days. Concurrently with the initiation of NaCl exposure, another set of seedlings received a foliar application of either 2 mM salicylic acid (SA) or 2 mM paclobutrazol (PBZ). *A.t.*, *Arabidopsis thaliana*; *E.s.*, *Eutrema salsugineum*; FW, fresh weight. Error bars indicate the mean ± SD of three biological replicates from independent plants, and different lowercase letters indicate significant differences at the 0.05 (*p* < 0.05) level.

Under control conditions, SA concentrations were four-fold lower in *E. salsugineum* than in *A. thaliana* (Figure 1). While NaCl treatment doubled SA levels in *A. thaliana*, it led to a decrease in *E. salsugineum*. This salt-induced SA accumulation in *A. thaliana* could be mimicked in *E. salsugineum* by exogenous application of 2 mM SA under stress. Conversely, treatment with 2 mM of the SA biosynthesis inhibitor PBZ [21] suppressed approximately 70% of SA accumulation.

The SA derivative methyl salicylate (MeSA) acts as a long-distance phloem-mobile signal for systemic resistance against various biotic and abiotic stresses [22,23]. It is synthesized from SA by SAMT. According to SSH results, salt stress promoted *SAMT* gene expression in *A. thaliana* but not in *E. salsugineum*. Correspondingly, basal MeSA levels were fourfold higher in control *A. thaliana* compared to *E. salsugineum* (Figure 1). NaCl treatment tripled MeSA concentrations in *A. thaliana* but had no significant effect on MeSA levels in *E. salsugineum*.

**Figure 2:**
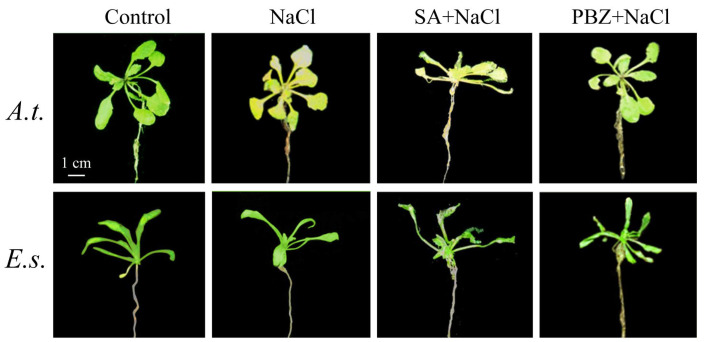
21-day-old seedlings under 3-day SA, PBZ, and NaCl treatments. *A.t.*, *Arabidopsis thaliana*; *E.s.*, *Eutrema salsugineum*.

NaCl treatment induced apparentwiltingand yellowing in *A. thaliana* but not in *E. salsugineum*. Co-treatment with SA exacerbated wilting and yellowing, while co-treatment with PBZ partially alleviated salt-induced wilting and yellowing (Figure 2).

**Figure 3:**
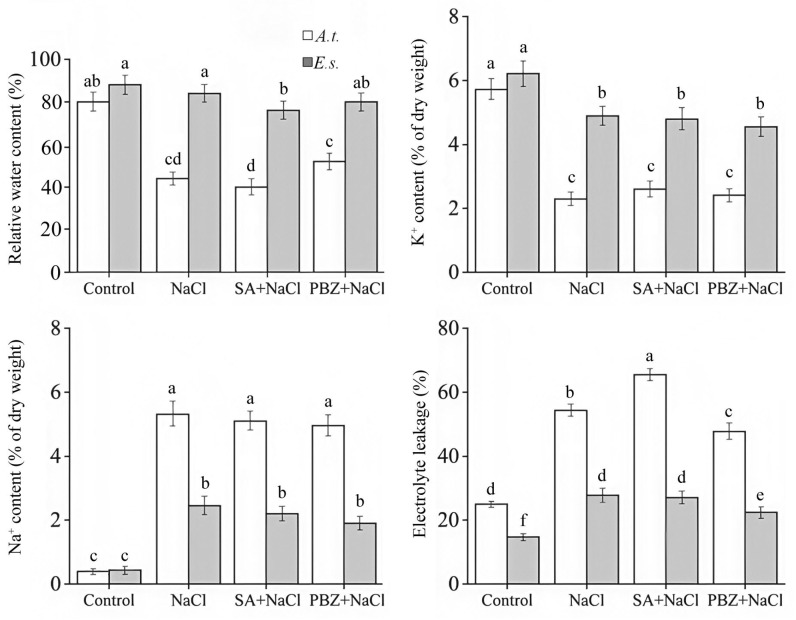
The effects of SA, PBZ, and NaCl treatments on relative water content, electrolyte leakage, K^+^, and Na^+^ contents. *A.t.*, *Arabidopsis thaliana*; *E.s.*, *Eutrema salsugineum*. Error bars indicate the mean ± SD of three biological replicates from independent plants, and different lowercase letters indicate significant differences at the 0.05 (*p* < 0.05) level.

NaCl treatment significantly reduced the relative water content (RWC) in both species. Co-treatment with SA exacerbated this reduction, while co-treatment with PBZ partially alleviated the reduction (Figure 3). Correspondingly, relative electrolyte leakage—an indicator of membrane damage and permeability—was markedly elevated under salt stress (Figure 3). This increase was further aggravated by SA but mitigated by PBZ. All these physiological changes were more pronounced in *A. thaliana* than in *E. salsugineum*.

Regarding ion homeostasis, leaf Na^+^ content increased dramatically under salt stress. In contrast, K^+^ content decreased under salinity (Figure 3). Neither SA co-treatment nor PBZ co-treatment affected Na^+^ or K^+^ content (Figure 3), suggesting that SA depletion plays no role in K^+^ uptake or Na^+^ excretion.

### 2.3. SA Does Not Affect Salt Stress-Responsive Genes Specific to Eutrema

**Figure 4:**
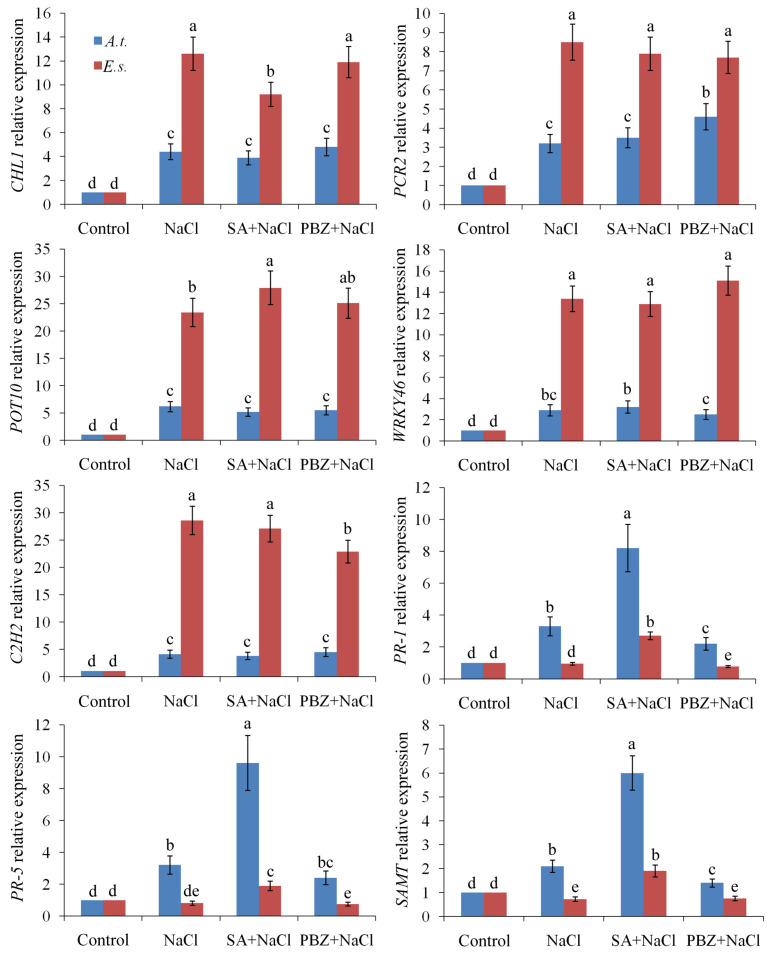
The effects of SA, PBZ, and NaCl treatments on eight representative salt stress -responsive genes. *A.t.*, *Arabidopsis thaliana*; *E.s.*, *Eutrema salsugineum*. The expression levels of the control seedlings were normalized to 1. Error bars indicate the mean ± SD of three biological replicates from independent plants, and different lowercase letters indicate significant differences at the 0.05 (*p* < 0.05) level.

To explore the mechanism by which SA depletion promotes salt stress tolerance, we analyzed salt-tolerant genes identified via SSH using q-PCR. Although all five representative salt stress-responsive genes specific to *Eutrema* (*CHL1*, *PCR2*, *POT10*, *WRKY46*, and *C2H2*) were induced by NaCl treatment (more pronouncedly in *E. salsugineum* than in *A. thaliana*), their expression was not significantly affected by either SA or PBZ (Figure 4). In contrast, the SA-signaling genes *PR-1* and *PR-2*, along with the SA-metabolism gene *SAMT*, were induced by either NaCl or SA treatment, with these responses being more pronounced in *A. thaliana* than in *E. salsugineum* (Figure 4). Therefore, SA depletion appears to enhance salinity tolerance independently of increased ionic homeostasis gene expression.

### 2.4. SA-Depletion Alleviates Oxidative Damage Under Salt Stress

**Figure 5:**
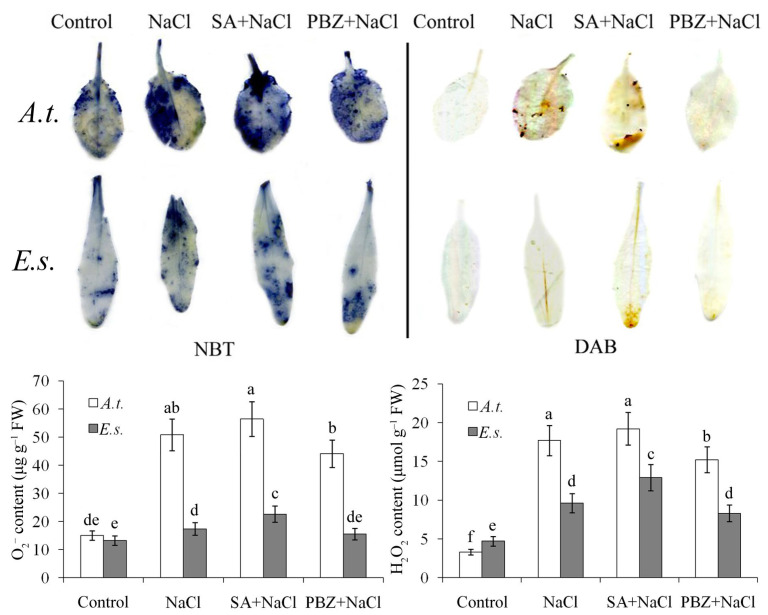
Oxidative damage parameters of seedlings under salt stress. O_2_^−^ or H_2_O_2_ deposition was stained by nitroblue tetrazolium (NBT) or 3,3-diaminobenzidine (DAB), respectively. *A.t.*, *Arabidopsis thaliana*; *E.s.*, *Eutrema salsugineum*. Quantitative detection results are shown below. Error bars indicate the mean ± SD of three biological replicates from independent plants, and different lowercase letters indicate significant differences at the 0.05 (*p* < 0.05) level.

Abiotic stresses like salinity can disrupt the cellular redox balance in plants. To investigate whether SA levels influence ROS accumulation under salt stress, we used nitroblue tetrazolium (NBT) and 3,3-diaminobenzidine (DAB) staining to detect superoxide (O_2_^−^) and hydrogen peroxide (H_2_O_2_) deposition, respectively (Figure 5). NaCl treatment intensified staining in both species, indicating elevated oxidative damage; however, *E. salsugineum* exhibited significantly less ROS accumulation than *A. thaliana*. SA co-treatment increased ROS staining, while PBZ co-treatment reduced it (Figure 4). These results are consistent with the electrolyte leakage data presented in Figure 3.

### 2.5. SA-Depletion Alleviates Oxidative Damage by Promoting Non-Enzymatic Antioxidants

The activities of four representative antioxidant enzymes—superoxide dismutase (SOD), peroxidase (POD), catalase (CAT), and ascorbate peroxidase (APX)—were activated under salt stress (Figure S1). However, neither SA nor PBZ significantly affected their activities. In some cases (e.g., POD and APX), both SA and PBZ treatments slightly inhibited enzyme activity (Figure S1).

**Figure 6:**
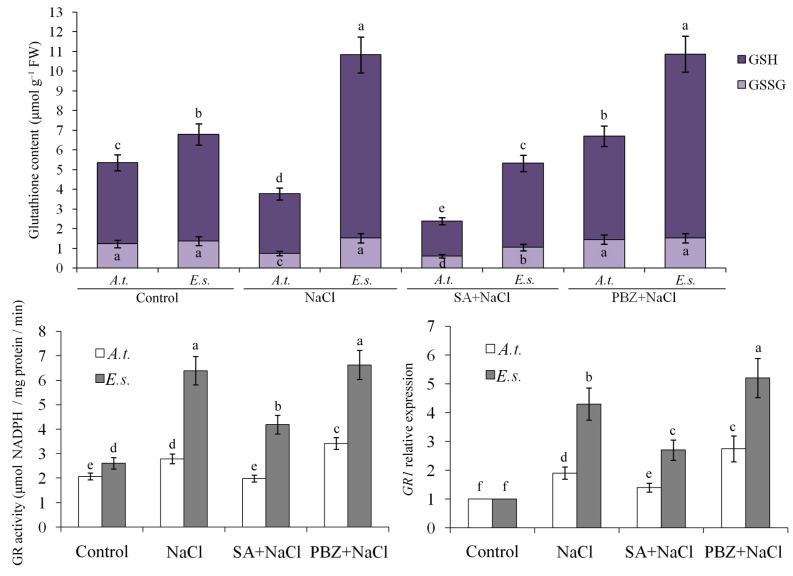
The effects of SA, PBZ, and NaCl treatments on glutathione content, GR activity, and *GR1* gene expression. *A.t.*, *Arabidopsis thaliana*; *E.s.*, *Eutrema salsugineum*; FW, fresh weight; GSH, reduced glutathione; GSSG, oxidized glutathione. The *GR1* gene expression levels of the control seedlings were normalized to 1. Error bars indicate the mean ± SD of three biological replicates from independent plants, and different lowercase letters indicate significant differences at the 0.05 (*p* < 0.05) level.

**Figure 7:**
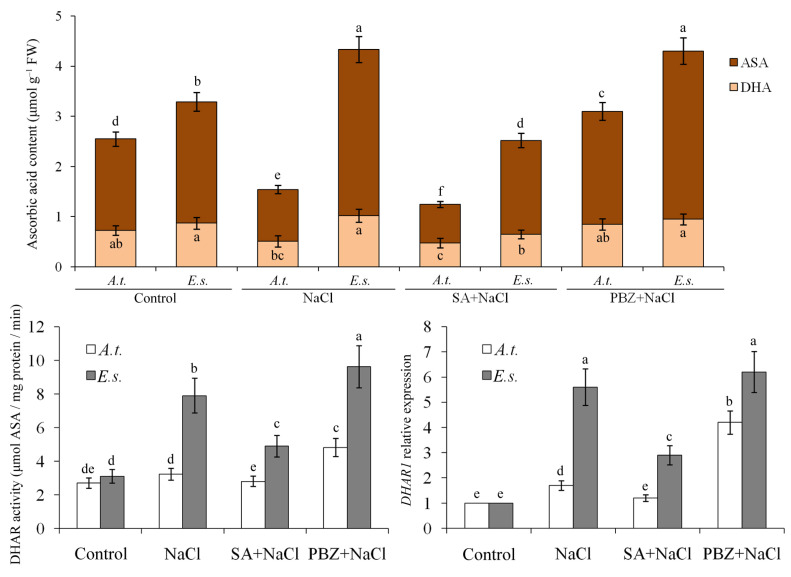
The effects of SA, PBZ, and NaCl treatments on ascorbic acid content, DHAR activity, and *DHAR1* gene expression. *A.t.*, *Arabidopsis thaliana*; *E.s.*, *Eutrema salsugineum*; FW, fresh weight; ASA, reduced ascorbic acid; DHA, dehydroascorbate. The *DHAR1* gene expression levels of the control seedlings were normalized to 1. Error bars indicate the mean ± SD of three biological replicates from independent plants, and different lowercase letters indicate significant differences at the 0.05 (*p* < 0.05) level.

We also measured the levels of GSH, GSSG, ASA, and DHA. Under 200 mM NaCl stress, glutathione (especially the reduced form) and ascorbic acid (especially the reduced form) increased in *E. salsugineum* but decreased in *A. thaliana* (Figure 6 and Figure 7). SA treatment reduced these antioxidants in both species. In contrast, PBZ treatment maintained significantly higher levels of GSH and ASA under the same stress conditions (Figure 6 and Figure 7).

Notably, the activities and corresponding gene expression of GR and DHAR increased significantly at 200 mM NaCl, both with and without 2 mM PBZ, but decreased slightly upon SA treatment. This pattern corresponds to the higher GSH/GSSG and ASA/DHA ratios observed at 200 mM NaCl with or without PBZ. These changes were more pronounced in *E. salsugineum* than in *A. thaliana* (Figure 6 and Figure 7). Overall, endogenous SA levels were negatively correlated with GR and DHAR activity and gene expression, suggesting that SA depletion alleviates oxidative damage by promoting non-enzymatic antioxidants at both the transcriptional and enzymatic levels.

## 3. Discussion

SA is a key signaling molecule with multiple functions in plants. In addition to its established role in pathogen defense, SA mediates responses to abiotic stress [24]. Research indicates that elevated SA concentrations typically heighten plant sensitivity to such stress, with most species exhibiting optimal stress tolerance at SA levels between 0.1 mM and 0.5 mM [24]. Maintaining an appropriate SA concentration is critical because it helps regulate sodium (Na^+^) uptake by roots and its translocation to shoots, while also inhibiting potassium (K^+^) efflux through depolarization-activated outward-rectifying K^+^ channels and ROS-activated non-selective cation channels [25,26]. In the present study, *Eutrema salsugineum* was found to contain four times less SA than *Arabidopsis thaliana* under control conditions. Salt (NaCl) treatment increased SA levels twofold in *A. thaliana* but not in *E. salsugineum*. Applying 2 mM SA exogenously to *E. salsugineum* raised its internal SA to match that of salt-stressed *A. thaliana*; at this concentration, SA increased oxidative injury in both species.

*E. salsugineum* exhibits a unique transcriptional response to salinity. Using suppression–subtractive hybridization, this study identified several salt-responsive genes specific to *Eutrema*. Among these, *CHL1* has not been previously linked to salt stress, but it functions upstream in glutathione-dependent phytochelatin biosynthesis under cadmium exposure [27] and influences zinc accumulation via a nitrate-dependent pathway [28]. Overexpression of *PCR2* enhances tolerance to cadmium and other heavy metals [29,30]. High-affinity potassium transporters, such as the POT10 protein, are essential for potassium acquisition. Various potassium transporters promote salt tolerance by modulating K^+/^Na^+^ homeostasis under saline conditions [31,32]. The transcription factor WRKY46 controls a network of genes related to cellular redox balance and stomatal aperture during salt stress [33]. Furthermore, WRKY46 is involved in a feed-forward loop that inhibits lateral root growth under osmotic/salt stress by influencing ABA signaling and auxin homeostasis [34,35]. Additional studies have shown that certain C2H2-type zinc finger proteins enhance salt tolerance by supporting ionic homeostasis and osmotic adjustment [36,37,38].

The application of SA and PBZ did not significantly alter the expression of the aforementioned genes. Consequently, the enhanced salinity tolerance observed in plants with depleted SA appears to operate independently of increased ionic homeostasis gene expression. This aligns with prior research demonstrating that SA-deficient Arabidopsis expressing a salicylate hydroxylase (*NahG*) gene exhibits greater tolerance to moderate salt stress [39]. A contributing factor may be the higher GSH/GSSG and ASA/DHA ratios maintained in *NahG* plants under stress, as reported previously [40]. Although these plants do not inherently produce more active antioxidant enzymes than wild-type plants under normal conditions, they sustain higher activities of GR and DHAR during stress [40]. Supporting this, salinity induced an increased pool of reduced ascorbate and total glutathione in *Eutrema*, but not in *Arabidopsis* [16]. Consistent with these findings, the present study observed that SA decreased, while PBZ increased, the contents of glutathione and ascorbic acid, the GSH/GSSG and ASA/DHA ratios, and the activities/gene expression of GR and DHAR. Therefore, the SA-depletion-induced rise in non-enzymatic antioxidants is likely a key mechanism behind *Eutrema*’s high salt stress tolerance.

The redox-sensitive transcriptional coactivator NPR1, which is central to SA signaling [25,26], may be involved. Further investigation is required to determine if NPR1 can bind to cis-acting elements or interact with trans-acting factors on the promoters of *GR* and *DHAR* genes to repress their transcription. Alternatively, SA depletion might regulate these genes through crosstalk with other phytohormones. For example, salt treatment induces a twofold increase in ABA in *A. thaliana*, but not in *E. salsugineum* [15]. In *E. salsugineum*, levels of jasmonic acid (JA) and jasmonoyl-L-isoleucine (JA-Ile) are low and further decrease under salinity [15]. These distinct hormonal responses may be linked to SA depletion in *Eutrema*. As indicated by Ismail et al. [41], the interaction between ABA and JA is particularly critical for determining plant survival under salt stress. More detailed mechanistic studies are needed to elucidate these pathways.

## 4. Materials and Methods

### 4.1. Plant Material and Growth Conditions

Seeds of *Arabidopsis thaliana* ecotype Col-0 were imbibed in a 2 mg/L gibberellin solution and subjected to a 2-day vernalization period at 4 °C in darkness. In contrast, *Eutrema salsugineum* ecotype Shandong seeds (a gift from Shandong Normal University, China) received the same gibberellin treatment but were vernalized for 30 days under identical dark, 4 °C conditions. Following stratification, all seeds were sown in vermiculite-filled pots. Seedlings were cultivated in a growth room set to 23 ± 1 °C, 70% humidity, a light intensity of 100 μmoL m^−2^ s^−1^, and a 16/8-h light/dark photoperiod.

For salt stress induction, 21-day-old seedlings were transferred to a half-strength MS nutrient solution supplemented with 200 mM NaCl for three days. Concurrently with the initiation of NaCl exposure, another set of seedlings received a foliar application of either 2 mM salicylic acid (SA) or 2 mM paclobutrazol (PBZ).

### 4.2. Two-Round Suppression Subtractive Hybridization (SSH)

Total RNA extraction was performed with TRIzol Reagent^®^ (Molecular Research Center, Cincinnati, OH, USA). For first-strand cDNA synthesis, 1 μg of total RNA was combined with 10 pmol of an anchor oligo dT primer (CDS-PT, containing an *Rsa* I site) in 10 μL of RNase-free water, heated to 70 °C for 10 min, and then cooled on ice for 5 min. This annealed mixture was combined with 200 U of PowerScript™ Reverse Transcriptase (Clontech, Mountain View, CA, USA) in a 20 μL reaction containing 1× first-strand buffer, 10 mM DTT, 10 pmol of a T-S primer (AD-G, with an *Rsa* I site) [42], and 1 mM each dNTP, followed by incubation at 42 °C for 90 min.

To generate the necessary quantity of driver cDNA for subtraction hybridization, aliquots (1–2 μL) of the first-strand cDNA were amplified using a 2× EasyTaq PCR SuperMix system (TransGen Biotech. Co., Ltd., Beijing, China) in a 50 μL reaction with the T-S PCR primer (AD-PCR). The cycling parameters were: 95 °C for 1 min, followed by 28–36 cycles of 95 °C for 30 s, 58 °C for 30 s, and 72 °C for 2.5 min. This approach, based on the SMARTer™ PCR cDNA Synthesis Kit (Clontech) template-switching method, yields over 2 μg of double-stranded cDNA from a small amount of starting RNA [43]. The amplified cDNA was purified with an E.Z.N.A^®^ Cycle-Pure Kit (Omega Biotech Corporation, Victoria, BC, Canada) and quantified.

Subsequent suppression–subtractive hybridization (SSH) was conducted according to standard protocols [43], utilizing the PCR-Select™ cDNA Subtraction Kit (Clontech) for convenience. The procedure involved sequential steps of Rsa I digestion, adaptor ligation to tester cDNA, first and second hybridizations, and suppression nested PCR amplification [43]. For a secondary SSH, 2 μg of purified product from the first SSH was used as both driver and tester cDNA, following the same standard procedures [43]. All primers used are listed in Table S1.

Four total RNA samples from whole seedlings were prepared: (I) *A. thaliana* seedlings before treatment; (II) *A. thaliana* seedlings after treatment; (III) *E. salsugineum* seedlings before treatment; (IV) *E. salsugineum* seedlings after treatment. Subtracting Sample I from Sample II yielded salt-induced genes in *A. thaliana* (Result “A.t.”), while subtracting Sample III from Sample IV yielded salt-induced genes in *E. salsugineum* (Result “E.s.”). Subtracting Result “A.t.” from Result “E.s.” produced a final set of salt-tolerance genes specific to *E. salsugineum*, whereas subtracting Result “E.s.” from Result “A.t.” yielded salt-tolerance genes specific to *A. thaliana*.

Following the secondary SSH, the resulting PCR products were cloned into the pEASY-T1 Simple vector (TransGen Biotech. Co., Ltd., Beijing, China). Positive clones were identified by blue/white screening, and 50 random positive clones from each product were selected for sequencing.

### 4.3. Quantitative Real-Time PCR Analysis

Gene expression was quantified via quantitative RT-PCR (q-PCR) with SYBR Premix Ex Taq (Takara Biomedical Technology, Dalian, China). The initial copy number of each target gene was derived from its threshold cycle (Ct), which is the PCR cycle at which the fluorescence signal rises above the background [44]. Each sample was analyzed with three biological replicates, and *ACTIN7* was used as an internal reference gene. Expression levels in *A. thaliana* or *E. salsugineum* seedlings under control (no NaCl) conditions were set to a value of 1. All primer sequences are provided in Table S2.

### 4.4. Salicylate Determination

Leaf samples for salicylate analysis were collected during midday. The procedures for extraction and analysis followed established methods by Dobrev and Kamínek [45] and Dobrev and Vankova [46]. In brief, fresh samples (approximately 100 mg) were homogenized and extracted using a methanol/water/formic acid mixture (15/4/1, *v*/*v*/*v*). Labeled internal standards—specifically ^2^H_4_-SA (Olchemim, Olomouc, Czech Republic) and ^2^H_4_-MeSA (CDN Isotopes, Quebec, QC, Canada)—were introduced at a concentration of 10 pmol per sample. Subsequent purification employed a C18 solid-phase extraction column (SepPak-C18, Waters, Milford, MA, USA), followed by separation on a reverse-phasecation-exchange column (Oasis-MCX, Waters). The hormone fraction was eluted with methanol and separated via HPLC (Model 1260, Agilent, Santa Clara, CA, USA). Finally, SA and MeSA were quantified from the crude plant extracts using HPLC mass spectrometry as described by Pan et al. [47].

### 4.5. Sodium and PotassiumContent Determination

Potassium content was quantified according to the method of [48] using a GC flame photometric detector (Agilent). For analysis, 0.5 g of fresh plant tissue underwent wet digestion with 6 mL of concentrated 98% H_2_SO_4_ and 3 mL of H_2_O_2_ over a 5 h period. The resulting mineralized digest was dissolved in 5 mL of 0.1 M HNO_3_, filtered through a 0.25 μm nylon membrane, and brought to a final volume of 100 mL with distilled water. A 5 mL aliquot of this solution was subsequently diluted to 50 mL. The concentrations of Na^+^ and K^+^ in the final diluted solution were determined via flame photometry, and the ion content in the original tissue was calculated using standard calibration curves.

### 4.6. ROS Staining and Quantification of Oxidative Damages

The accumulation of superoxide anions (O_2_^−^) and hydrogen peroxide (H_2_O_2_) in leaves was initially assessed through histochemical staining, as outlined by [49]. Leaf segments were incubated for 3 h in either 0.8 mg/mL nitroblue tetrazolium (NBT) or 2.4 mg/mL 3,3-diaminobenzidine (DAB). Following destaining in 75% ethanol, the samples were imaged and archived using a Leica M 165 C/FC stereomicroscope.

For quantitative analysis, the contents of H_2_O_2_ and O_2_^−^ were measured following an established protocol [50]. Leaves (0.5 g) were harvested three days post-stress treatment, ground in liquid nitrogen, and homogenized in 5 mL of 0.1% (*w*/*v*) trichloroacetic acid (TCA). After centrifugation at 12,000×*g* for 20 min at 4 °C, 1 mL of the supernatant was mixed with 0.5 mL of 10 mM sodium phosphate buffer (pH 7.0) and 1 mL of 1 M potassium iodide (KI). The H_2_O_2_ concentration was determined by measuring absorbance at 390 nm.

To quantify O_2_^−^, 0.5 g of leaf tissue was powdered in 1.5 mL of 65 mM sodium phosphate buffer (pH 7.8) and centrifuged at 10,000× *g* for 15 min. A 0.5 mL aliquot of the supernatant was combined with 0.5 mL of the same phosphate buffer and 0.1 mL of 10 mM hydroxylamine hydrochloride. The mixture was incubated at 25 °C for 20 min. Subsequently, 1 mL of 58 mM p-aminobenzenesulfonic acid and 1 mL of 7 mM α-naphthylamine were added, followed by a further 20 min incubation at 25 °C. The final reaction mixture was extracted with an equal volume of chloroform, and the absorbance of the aqueous phase was read at 530 nm to determine O_2_^−^ content.

Electrolyte leakage was evaluated using the method of Dionisio-Sese and Tobita [51]. After detecting the conductivity, the plant sample was boiled for 20 min to achieve 100% electrolyte leakage. Relative water content was determined as the ratio of (fresh mass − dry mass)/(water-saturated mass − dry mass) [52].

### 4.7. Determination of Antioxidant Enzyme Activities

Superoxide dismutase (SOD) activity was quantified by measuring the ability to inhibit the photochemical reduction of nitroblue tetrazolium following the method of Liu et al. [53]. For peroxidase (POD) activity assessment, the method of Verma and Mishra [54] was followed. The amount of H_2_O_2_ utilized was extrapolated from the standard curve between A_520_ and H_2_O_2_ concentration. Catalase (CAT) was measured as the decline in absorbance at 240 nm due to the decrease in extinction of H_2_O_2_, and ascorbate peroxidase (APX) activities were determined by the decrease in absorbance at 290 nm as ascorbate was oxidized according to the procedures described by Esfandiari et al. [55]. Dehydroascorbate reductase (DHAR) activity was evaluated by monitoring the rise in absorbance at 265 nm, corresponding to reduced ascorbate formation [40]. Glutathione reductase (GR) activity was measured based on the decline in NADPH absorbance at 340 nm [40].

### 4.8. Determination of GSH, GSSG, ASA, and DHA

The extraction and quantification of reduced ascorbic acid (ASA) and dehydroascorbate (DHA) were performed according to established methods [56,57]. Fresh leaf tissue (250 mg) was homogenized in 2.0 mL of 10% (*w*/*v*) trichloroacetic acid (TCA) and centrifuged for 5 min at 10,000× *g* total ascorbate (after reducing DHA to ASA by dithiothreitol), and ASA in the supernatant, were measured as Fe^2+^–bipyridine complex (absorbance maximum at 525 nm) that was formed after reduction of Fe^3+^ by ASA. DHA content was calculated from the difference between total ascorbate and ASA.

Similarly, the procedures for extracting and determining reduced glutathione (GSH) and oxidized glutathione (GSSG) followed previously described protocols [57,58]. For the GSSG assay, 40 mM N-ethylmaleimide (NEM) dissolved in 10 mM sodium-phosphate buffer (pH 7.5) containing 5 mM EDTA was added to the supernatant for masking the thiol group of GSH by NEM. After mixing, TCA and NEM were removed by extracting three times with diethylether. Glutathione was determined by the DTNB [5,5′-dithiobis(2-nitrobenzoic acid)]-recycling method. An increase in absorbance at 412 nm was measured after the addition of DTNB.

### 4.9. Data Analysis

All experiments were conducted with three biological replicates from independent plants, with two technical replicates per plant, and results are expressed as the mean ± standard deviation (*n* = 3). Statistical significance was evaluated by one-way analysis of variance (ANOVA) followed by Tukey’s post hoc test for multiple comparisons, with a threshold of *p* < 0.05 considered significant.

## Figures and Tables

**Table 1:** Salt stress-responsive genes specific to *Arabidopsis*.

Gene ID	Description	Frequency
At1g16030	Heat shock protein 70b	2/50
At4g25200	23.6 kDa heat shock protein in mitochondria	1/50
At4g31500	Cytochrome P450 94B1	2/50
At5g52300	Desiccation-responsive protein 29B	1/50
At4g36040	Chaperone protein dnaJ 11 (DNAJ heat shock protein)	3/50
At5g11260	Long Hypocotyl 5 (HY5; bZIP transcription factor 56)	2/50
At1g69010	Basic helix-loop-helix protein 125 (bHLH125)	4/50
At3g62420	Homeobox-leucine zipper protein ATHB-53 (bZIP53)	1/50
At5g51490	pectinesterase/pectinesterase inhibitor	3/50
At5g53210	Transcription factor SPEECHLESS	1/50
At5g05880	UDP-glycosyltransferase 76C4	2/50
At1g63700	Mitogen-activated protein kinase kinasekinase YODA	1/50
At4g04950	Monothiol glutaredoxin-S17 (GrxS17)	2/50
At1g80820	Cinnamoyl-CoA reductase 2	4/50
At5g38020	**Salicylate/benzoate carboxyl methyltransferase (SAMT)**	2/50
At2g14610	**Pathogenesis-related protein PR-1**	3/50
At1g75040	**Pathogenesis-related protein PR-5**	1/50
At1g20450	Dehydrin ERD10 (Low-temperature-induced protein LTI45)	5/50
At4g37990	Cinnamyl alcohol dehydrogenase 8 (CAD8)	2/50
At2g44790	Uclacyanin-2 (Blue copper-binding protein II)	3/50
At5g21040	EIN3-binding F-box protein 2 (FBX2)	2/50
At2g36870	xyloglucan endotransglucosylase/hydrolase protein 32 (XTH32)	2/50
At4g17490	Ethylene-responsive transcription factor (ERF6)	1/50

50 random positive clones for each PCR product were sequenced. Genes in bold are related to SA metabolism or signaling and verified by q-PCR. “Frequency” refers to the number of times the same gene sequencing result appears among 50 positive clones.

**Table 2:** Salt stress-responsive genes specific to *Eutrema*.

Gene ID	Description	Frequency
At2g07707	Plant mitochondrial ATPase, F0 complex	2/50
**At1g12110**	**NPK1-related protein kinase 2 chlorate/nitrate transporter (CHL1)**	6/50
At4g38460	Geranyl pyrophosphate synthase-related protein (GGR)	2/50
At2g36390	Starch branching enzyme class II (SBE2-1)	3/50
**At2g46400**	**WRKY transcription factor 46**	1/50
At1g01160	GRF1-interacting factor 2 (GIF2)	3/50
At5g09660	Microbody NAD-dependent malate dehydrogenase	2/50
**At4g17810**	**C2H2 zinc fingers superfamily protein**	2/50
At3g59970	Methylenetetrahydrofolate reductase MTHFR1	2/50
At3g15190	F4B12_10 mRNA, chloroplast 30S ribosomal protein S20	4/50
At4g38740	Peptidylprolyl isomerase ROC1	5/50
At1g50940	U50582 putative electron transport flavoprotein	3/50
At5g11520	Aspartate aminotransferase ASP3	3/50
At4g38970	Fructose-bisphosphate aldolase-like protein	2/50
At5g11060	Putative homeobox protein knotted-1 Like 4 (KNat4)	2/50
At4g11570	Haloacid dehalogenase-like hydrolase (HAD) superfamily protein	3/50
**At1g14870**	**A membrane protein involved in zinc transport and detoxification (Plant Cadmium Resistance 2; PCR2)**	2/50
At3g57010	Strictosidine synthase	1/50
**At1g31120**	**K^+^ uptake permease 10 (Potassium transporter 10; POT10)**	2/50

50 random positive clones for each PCR product were sequenced. Genes in bold are related toionic homeostasis directly or indirectly and verified by q-PCR. “Frequency” refers to the number of times the same gene sequencing result appears among 50 positive clones.

## Data Availability

All data generated or analyzed during this study are included in this published article and its Supplementary Information File.

## Supplementary Materials

The following supporting information can be downloaded at: https://www.mdpi.com/article/10.3390/ijms27031168/s1.

## References

[1] Melino V., Tester M. (2023). Salt-tolerant crops: Time to deliver. Annu. Rev. Plant Biol..

[2] Raza A., Zaman Q.U., Shabala S., Tester M., Munns R., Hu Z., Varshney R.K. (2025). Genomics-assisted breeding for designing salinity-smart future crops. Plant Biotechnol. J..

[3] Garcia-Daga S., Roy S.J., Gilliham M. (2025). Redefining the role of sodium exclusion within salt tolerance. Trends Plant Sci..

[4] Tripathi J.M., Khan B.R., Gaur R., Yadav D., Verma K.K., Gupta R. (2025). Gibberellic acid improves photosynthetic electron transport and stomatal function in crops that are adversely affected by salinity exposure. Plants.

[5] Basu S., Kumar G. (2024). Regulation of nitro-oxidative homeostasis: An effective approach to enhance salinity tolerance in plants. Plant Cell Rep..

[6] Kazachkova Y., Eshel G., Pantha P., Cheeseman J.M., Dassanayake M., Barak S. (2018). Halophytism: What have we learnt from *Arabidopsis thaliana* relative model systems?. Plant Physiol..

[7] Li C., Duan C., Zhang H., Zhao Y., Meng Z., Zhao Y., Zhang Q. (2022). Adaptative mechanisms of halophytic *Eutremasalsugineum* encountering saline environment. Front. Plant Sci..

[8] Gong Q., Li P., Ma S., Indu Rupassara S., Bohnert H.J. (2005). Salinity stress adaptation competence in the extremophile *Thellungiella halophila* in comparison with its relative *Arabidopsis thaliana*. Plant J..

[9] Wong C.E., Li Y., Labbe A., Guevara D., Nuin P., Whitty B., Diaz C., Golding G.B., Gray G.R., Weretilnyk E.A. (2006). Transcriptional profiling implicates novel interactions between abiotic stress and hormonal responses in *Thellungiella*, a close relative of *Arabidopsis*. Plant Physiol..

[10] Lee Y.P., Giorgi F.M., Lohse M., Kvederaviciute K., Klages S., Usadel B., Meskiene I., Reinhardt R., Hincha D.K. (2013). Transcriptome sequencing and microarray design for functional genomics in the extremophile Arabidopsis relative *Thellungiellasalsuginea* (*Eutremasalsugineum*). BMC Genom..

[11] Li C., Qi Y., Zhao C., Wang X., Zhang Q. (2021). Transcriptome profiling of the salt stress response in the leaves and roots of halophytic *Eutremasalsugineum*. Front. Genet..

[12] Pang Q., Chen S., Dai S., Chen Y., Wang Y., Yan X. (2010). Comparative proteomics of salt tolerance in *Arabidopsis thaliana* and *Thellungiella halophila*. J. Proteome Res..

[13] Arbona V., Argamasilla R., Gómez-Cadenas A. (2010). Common and divergent physiological, hormonal and metabolic responses of *Arabidopsis thaliana* and *Thellungiella halophila* to water and salt stress. J. Plant Physiol..

[14] Athar H.U., Zulfiqar F., Moosa A., Ashraf M., Zafar Z.U., Zhang L., Ahmed N., Kalaji H.M., Nafees M., Hossain M.A. (2022). Salt stress proteins in plants: An overview. Front. Plant Sci..

[15] Pilarska M., Wiciarz M., Jajić I., Kozieradzka-Kiszkurno M., Dobrev P., Vanková R., Niewiadomska E. (2016). A different pattern of production and scavenging of reactive oxygen species in halophytic *Eutremasalsugineum* (*Thellungiellasalsuginea*) plants in comparison to *Arabidopsis thaliana* and its relation to salt stress signaling. Front. Plant Sci..

[16] Wiciarz M., Niewiadomska E., Kruk J. (2018). Effects of salt stress on low molecular antioxidants and redox state of plastoquinone and P700 in *Arabidopsis thaliana* (glycophyte) and *Eutremasalsugineum* (halophyte). Photosynthetica.

[17] Zhou M., Ghnaya T., Dailly H., Cui G., Vanpee B., Han R., Lutts S. (2019). The cytokinin trans-zeatine riboside increased resistance to heavy metals in the halophyte plant species *Kosteletzkyapentacarpos* in the absence but not in the presence of NaCl. Chemosphere.

[18] Kreps J.A., Wu Y., Chang H.S., Zhu T., Wang X., Harper J.F. (2002). Transcriptome changes for Arabidopsis in response to salt, osmotic, and cold stress. Plant Physiol..

[19] Yuan S., Zhang Z.W., Zheng C., Zhao Z.Y., Wang Y., Feng L.Y., Niu G., Wang C.Q., Wang J.H., Feng H. (2016). Arabidopsis cryptochrome 1 functions in nitrogen regulation of flowering. Proc. Natl. Acad. Sci. USA.

[20] Zhang Z.W., Feng L.Y., Wang J.H., Fu Y.F., Cai X., Wang C.Q., Du J.B., Yuan M., Chen Y.E., Xu P.Z. (2019). Two-factor ANOVA of SSH and RNA-seq analysis reveal development-associated Pi-starvation genes in oilseed rape. Planta.

[21] Xiang Y., Song M., Wei Z., Tong J., Zhang L., Xiao L., Ma Z., Wang Y. (2011). A jacalin-related lectin-like gene in wheat is a component of the plant defence system. J. Exp. Bot..

[22] Zhu F., Xi D.H., Yuan S., Xu F., Zhang D.W., Lin H.H. (2014). Salicylic acid and jasmonic acid are essential for systemic resistance against *tobacco mosaic virus* in *Nicotiana benthamiana*. Mol. Plant Microbe Interact..

[23] Gao W., Liu Y., Huang J., Chen Y., Chen C., Lu L., Zhao H., Men S., Zhang X. (2021). MES7 modulates seed germination via regulating salicylic acid content in Arabidopsis. Plants.

[24] Yuan S., Lin H.H. (2008). Role of salicylic acid in plant abiotic stress. Z. Naturforsch. C.

[25] Jayakannan M., Bose J., Babourina O., Shabala S., Massart A., Poschenrieder C., Rengel Z. (2015). The NPR1-dependent salicylic acid signalling pathway is pivotal for enhanced salt and oxidative stress tolerance in Arabidopsis. J. Exp. Bot..

[26] Seo S., Kim Y., Park K. (2023). NPR1 translocation from chloroplast to nucleus activates plant tolerance to salt stress. Antioxidants.

[27] Lu Y., Li T., Li R., Zhang P., Li X., Bai Z., Wu J. (2024). Role of SbNRT1.1B in cadmium accumulation is attributed to nitrate uptake and glutathione-dependent phytochelatins biosynthesis. J. Hazard. Mater..

[28] Pan W., You Y., Weng Y.N., Shentu J.L., Lu Q., Xu Q.R., Liu H.J., Du S.T. (2020). Zn stress facilitates nitrate transporter 1.1-mediated nitrate uptake aggravating Zn accumulation in Arabidopsis plants. Ecotoxicol. Environ. Saf..

[29] Reddy C.S., Cho M., Kaul T., Joeng J.T., Kim K.M. (2023). *Pseudomonas fluorescens* imparts cadmium stress tolerance in *Arabidopsis thaliana* via induction of AtPCR2 gene expression. J. Genet. Eng. Biotechnol..

[30] Menhas S., Hayat K., Lin D., Shahid M., Bundschuh J., Zhu S., Hayat S., Liu W. (2024). Citric acid-driven cadmium uptake and growth promotion mechanisms in *Brassica napus*. Chemosphere.

[31] Sustr M., Soukup A., Tylova E. (2019). Potassium in root growth and development. Plants.

[32] Templalexis D., Tsitsekian D., Daras G., Avgeri F., Kinoshita T., Gifford M.L., Hatzopoulos P., Rigas S. (2025). Cellular pH homeostasis shapes root system architecture by modulating auxin-mediated developmental responses. Plant Physiol..

[33] Ding Z.J., Yan J.Y., Xu X.Y., Yu D.Q., Li G.X., Zhang S.Q., Zheng S.J. (2014). Transcription factor WRKY46 regulates osmotic stress responses and stomatal movement independently in Arabidopsis. Plant J..

[34] Ding Z.J., Yan J.Y., Li C.X., Li G.X., Wu Y.R., Zheng S.J. (2015). Transcription factor WRKY46 modulates the development of Arabidopsis lateral roots in osmotic/salt stress conditions via regulation of ABA signaling and auxin homeostasis. Plant J..

[35] Chang L., Liu S., Zhang N., Yang D., Liu Z., Yuan H., Yan R., Lan X., Yukawa Y., Wu J. (2025). OsR126 IncRNA integrates AtWRKY46 and AtEM1 into ABA signaling in Arabidopsis. Plant Cell Rep..

[36] Han G., Yuan F., Guo J., Zhang Y., Sui N., Wang B. (2019). AtSIZ1 improves salt tolerance by maintaining ionic homeostasis and osmotic balance in Arabidopsis. Plant Sci..

[37] Yu Z., Yan H., Liang L., Zhang Y., Yang H., Li W., Choi J., Huang J., Deng S. (2021). A C_2_H_2_-type zinc-finger protein from *Millettia pinnata*, MpZFP1, enhances salt tolerance in transgenic Arabidopsis. Int. J. Mol. Sci..

[38] Wang D.R., Yang K., Wang X., You C.X. (2022). A C2H2-type zinc finger transcription factor, MdZAT17, acts as a positive regulator in response to salt stress. J. Plant Physiol..

[39] Borsani O., Valpuesta V., Botella M.A. (2001). Evidence for a role of salicylic acid in the oxidative damage generated by NaCl and osmotic stress in Arabidopsis seedlings. Plant Physiol..

[40] Cao Y., Zhang Z.W., Xue L.W., Du J.B., Shang J., Xu F., Yuan S., Lin H.H. (2009). Lack of salicylic acid in Arabidopsis protects plants against moderate salt stress. Z. Naturforsch. C.

[41] Ismail A., Seo M., Takebayashi Y., Kamiya Y., Eiche E., Nick P. (2014). Salt adaptation requires efficient fine-tuning of jasmonatesignalling. Protoplasma.

[42] Huang J.C., Chen F. (2006). Simultaneous amplification of 5′ and 3′ cDNA ends based on template-switching effect and inverse PCR. BioTechniques.

[43] Diatchenko L., Lau Y.F., Campbell A.P., Chenchik A., Moqadam F., Huang B., Lukyanov S., Lukyanov K., Gurskaya N., Sverdlov E.D. (1996). Suppression subtractive hybridization: A method for generating differentially regulated or tissue-specific cDNA probes and libraries. Proc. Natl. Acad. Sci. USA.

[44] Czechowski T., Stitt M., Altmann T., Udvardi M.K., Scheible W.-R. (2005). Genome-wide identification and testing of superior reference genes for transcript normalization in *Arabidopsis*. Plant Physiol..

[45] Dobrev P.I., Kamínek M. (2002). Fast and efficient separation of cytokinins from auxin and abscisic acid and their purification using mixed-mode solid-phase extraction. J. Chromatogr. A.

[46] Dobrev P.I., Vankova R. (2012). Quantification of abscisic acid, cytokinin, and auxin content in salt-stressed plant tissues. Methods Mol. Biol..

[47] Pan X.Q., Welti R., Wang X.M. (2010). Quantitative analysis of major plant hormones in crude plant extracts by high-performance liquid chromatography–mass spectrometry. Nat. Protoc..

[48] Rezaeinejad R., Khademi H., Ayoubi S., Mosaddeghi M.R. (2021). Roots under water stress induce K release from phlogopite, bio-transforming to vermiculite. Rhizosphere.

[49] Xiao Y., Zhang Z.W., Yang X.Y., Xie L.B., Chen L.P., Chen Y.E., Yuan M., Chen G.D., Yuan S. (2025). Effect of singlet oxygen on the stomatal and cell wall of rice seedling under different stresses. Int. J. Mol. Sci..

[50] Chen Y.-E., Cui J.-M., Yang J.-C., Zhang Z.-W., Yuan M., Song C., Yang H., Liu H.-M., Wang C.-Q., Zhang H.-Y. (2015). Biomonitoring heavy metal contaminations by moss visible parameters. J. Hazard. Mater..

[51] Dionisio-Sese M.L., Tobita S. (1998). Antioxidant responses of rice seedlings to salinity stress. Plant Sci..

[52] Arndt S.K., Irawan A., Sanders G.J. (2015). Apoplastic water fraction and rehydration techniques introduce significant errors in measurements of relative water content and osmotic potential in plant leaves. Physiol. Plant..

[53] Liu X., Peng K., Wang A., Lian C., Shen Z. (2010). Cadmium accumulation and distribution in populations of *Phytolacca americana* L. and the role of transpiration. Chemosphere.

[54] Verma S., Mishra S.N. (2005). Putrescine alleviation of growth in salt stressed *Brassica juncea* by inducing antioxidative defense system. J. Plant Physiol..

[55] Esfandiari E., Shakiba M.R., Mahboob S.A., Alyari H., Toorchi M. (2007). Water stress, antioxidant enzyme activity and lipid peroxidation in wheat seedling. J. Food Agric. Environ..

[56] Kampfenkel K., Van Montagu M., Inzé D. (1995). Extraction and determination of ascorbate and dehydroascorbate from plant tissue. Anal. Biochem..

[57] Zhang Z.W., Deng Z.L., Tao Q., Peng H.Q., Wu F., Fu Y.F., Yang X.Y., Xu P.Z., Li Y., Wang C.Q. (2022). Salicylate and glutamate mediate different Cd accumulation and tolerance between *Brassica napus* and *B. juncea*. Chemosphere.

[58] Zhang Z.W., Dang T.T., Yang X.Y., Xie L.B., Chen Y.E., Yuan M., Chen G.D., Zeng J., Yuan S. (2024). γ-aminobutyric acid alleviates programmed cell death in two Brassica species under cadmium stress. Int. J. Mol. Sci..

