# Supplementary material for: Enhancement of Non-Enzymatic Antioxidants in Eutrema salsugineum Under Salt Stress Depends on Salicylate Depletion
Source: Int J Mol Sci. 2026 Jan 23;27(3):1168. doi: 10.3390/ijms27031168 (PMC12897984; doi:10.3390/ijms27031168)
Supplement: Supplementary file 1 [file ijms-27-01168-s001.zip › ijms-4081484-supplementary.pdf]

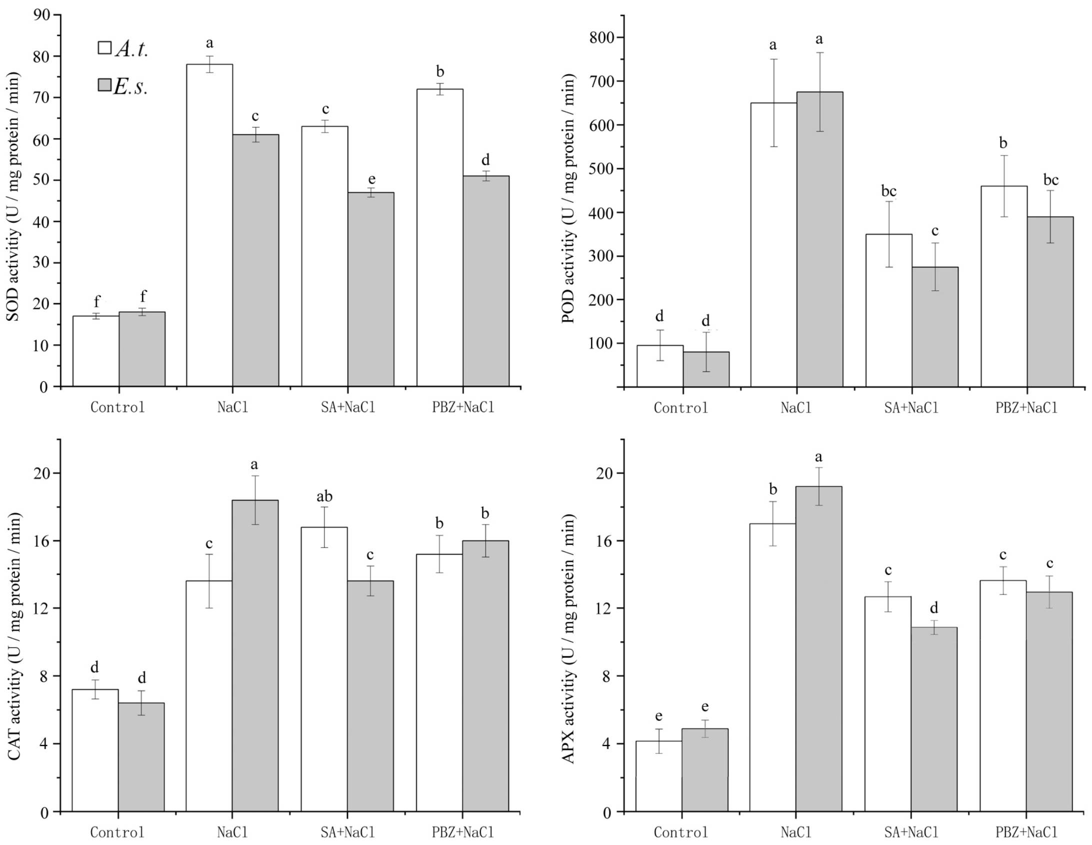

Figure S1. The effects of SA, PBZ and NaCl treatments on SOD, POD, CAT and APX activities. *A.t.*, *Arabidopsis thaliana*; *E.s.*, *Eutrema salsugineum*; U, units; Error bars indicate the mean ± SD of three biological replicates from independent plants, and different lowercase letters indicate significant differences at the 0.05 ($p < 0.05$) level.

Table S1. Adaptors and primers used for SSH.

Adaptor & Primer	Sequence
CDS-PT	AAGCAGTGGTATCAACGCAGAGTACTTTTTTTTTTTTTTTTTTTTTTTTT
AD-G	AAGCAGTGGTATCAACGCAGAGTACGGGGG
AD-PCR	AAGCAGTGGTATCAACGCAGAGTAC
Adaptor 1	5’-CTAATACGACTCACTATAGGGCTCGAGCGGCCGCCCGGGCAGGT-3’<br>3’-GGGCCCGTCCA-5’
Adaptor 2R	5’-CTAATACGACTCACTATAGGGCAGCGTGGTCGCGGCCGAGGT-3’<br>3’-GCCGGCTCCA-5’
PCR Primer 1	CTAATACGACTCACTATAGGGC
Nested PCR Primer 1	TCGAGCGGCCGCCCGGGCAGGT
Nested PCR Primer 2R	AGCGTGGTCGCGGCCGAGGT

Table S2. The primers used for RT-qPCR.

Gene name	Locus	Forward primer	Reverse primer
ACT7	At5g09810	ATGGTTGGTATGGGTCAGAAAGA	GACGAAGGATAGCATGAGGAAGA
CHL1	At1g12110	CGGCAGATCCGTCCTATCTCTA	ATTCGCACGATTTGTTTCACTT
PCR2	At1g14870	AAGGAGAATGGTCCACAGGCT	CACGGTATTGTTGGGTCAAAG
POT10	At1g31120	CCTCACACTCATCCCTCTCCTC	CCTATCACCATGCACGTACCAA
WRKY46	At2g46400	TCACATCTCCCACCAATCTCACT	TTTTCCACACACCGTTCTCAATC
C2H2	At4g17810	CCACCAACCCCATCTTCTTCTT	GCCAATGCTCTCATCATCTCCA
SAMT	At5g38020	CATGAAGGGAGGAGATGGAGAAC	AGCCAAAATGCCAAAATAGAGAG
PR-1	At2g14610	CGAACCCCAAAATCATAACACA	CGAGGGAAGAACAAGAGCACCT
PR-5	At1g75040	TCTTCCTCGTGTTCATCACAAGC	GAGTCAATTCAAATCCTCCATCG
GR1	At3g54660	TCTCAAACCACCGCTATTACCAC	TTTCCACGACCTTCAATCAACTT
DHAR1	At1g19570	ACTGGTGGGTGGAGAATGGTAA	GCAGGAGTCTTGAGTGGTGGAT
